# Implementation and Evaluation of a Standard Operating Procedure for Pediatric Infliximab Infusions

**DOI:** 10.1097/pq9.0000000000000137

**Published:** 2019-02-12

**Authors:** Maureen M. Kelly, Barbara S. Turner, Michael D. Kappelman, Eun Jeong Lee, Ajay S. Gulati

**Affiliations:** From the * Division of Pediatric Gastroenterology, UNC-Chapel Hill Hospitals, Chapel Hill, N.C.; † Duke University School of Nursing, Durham, N.C.

## Abstract

**Background:**

The purpose of this quality improvement project was to decrease care variation regarding infliximab delivery at a pediatric inflammatory bowel disease (IBD) center. This variation was driven by differences in provider and nurse practices within 2 distinct infliximab administration units in our center. Following the development of an infusion protocol, the primary project goal was to assess improvement in the submission and completion of a protocol-specific preinfusion safety checklist.

**Methods:**

The infusion protocol was developed based on the standard of care and expert physician opinion. A safety checklist was created to screen for active IBD symptoms and infection. We utilized continuous quality improvement to evaluate and guide the implementation of this preinfusion checklist. Checklist completion was assessed monthly over 15 months. We also conducted focus group interviews with infusion nurses and physicians to solicit qualitative protocol feedback.

**Results:**

We used standard run chart rules and identified a shift in the median completion rate for both units, with no trends or astronomical points. The baseline period was defined as the 6-month post-checklist implementation. The median baseline completion rate for 1 unit was 46%, and during the subsequent 9 months, the rate increased to 81%. In the other unit, the median baseline completion rate was 91%, and during the succeeding 9 months, the rate was 95%. Focus group feedback included themes of quality, communication, safety, and efficiency and helped improve the protocol.

**Conclusions:**

Feasibility was established for a standardized protocol to improve completion of a preinfusion safety checklist in children with IBD who receive infliximab. Nurse and physician focus group feedback was important for guiding protocol refinements.

## INTRODUCTION

Care of the pediatric patient with inflammatory bowel disease (IBD) has advanced significantly in recent years, including the routine use of biologic therapies such as infliximab (Remicade; Janssen Biotech, Horsham, PA) for moderate-to-severe IBD.^1–3^ In pediatric patients with IBD, infliximab improves symptoms, enhances growth,^4^ promotes mucosal healing,^5^ and has a favorable safety profile.^6–9^ In our practice, we treat approximately 40% of children with IBD with infliximab.

Although infliximab is generally safe and well-tolerated, it has known risks, including acute infusion reactions that occur in 3%–15% of pediatric patients.^10^ Other risks include viral, bacterial, and opportunistic fungal infections^1,3,10,11^; potential hepatotoxicity^12,13^; and possibly rare, long-term malignancy risk.^1,9,14^ Children with IBD who receive infliximab, therefore, need to be consistently monitored both clinically and with appropriate laboratory tests. Nonetheless, there are significant practice variations in infliximab utilization for pediatric patients with this high-risk disease.^10,15,16^ This practice variation is largely due to a lack of evidence-based guidelines regarding its administration and the management of adverse reactions.^10,17^ Other factors include adjustment of infusion doses, intervals, and other disease testing. Management is also based on symptoms and the results of laboratory screening that assesses nutritional status, intestinal inflammation, and drug toxicity.

This project was motivated by a perceived variation in practice among prescribing physicians and nurses in 2 distinct infusion units within our IBD center. Specific care variations included inconsistent use of a preinfusion safety checklist by nurses, different laboratory screening ordered by providers, diverse opinions regarding the need for premedications, and variability in the management of infusion reactions. Variations in care occurred both between and within locations. To address this variation, we developed an infliximab standard operating procedure (SOP) at our institution. In this study, our primary goal was to improve the submission and completion of a safety checklist associated with this SOP. Completion of protocol-specified laboratory testing was also assessed retrospectively, as a secondary measure of protocol fidelity.

## METHODS

We used the Standards for Quality Improvement Reporting Excellence 2.0 guidelines in the preparation of this article.^18^

### Setting

In our center, approximately 800 infliximab infusions are administered yearly (median of 64/mo; interquartile range of 59–74/mo) to approximately 150 pediatric patients in 2 infusion units: (a) The Children’s Short-Stay Unit (CSSU), providing specialty and postoperative care to day patients (N = 312 infusions/y); (b) The Infusion Room (IR), providing only infusions (N = 473 infusions/y). Each unit has a nurse manager and staff. Nurses have ≥1 year of experience and have completed a 6-week, infusion-specific orientation program.

### Interventions

We developed the SOP to establish a consistent approach to the administration of infliximab infusions. In addition to standardizing administration of infliximab, the SOP provided guidelines for utilization of a preinfusion safety checklist, preinfusion laboratory tests, and standardization of premedication (ie, prednisolone, diphenhydramine, etc). Standard laboratory tests included complete blood count, inflammatory markers, albumin, and aminotransferases. Ad hoc periodic tests included yearly 25-hydroxy vitamin D (vitamin D 25-OH) levels, tuberculosis screening, and fourth dose infliximab trough levels.

We developed the SOP based on a literature review,^3,10,15,17,19–24^ discussions with other pediatric IBD centers, and expert opinion. Before implementation, several stakeholders reviewed and approved the protocol. The reviewers included the prescribing physicians and nurses in the Division of Pediatric Gastroenterology, infusion nurses and managers, pharmacists, allergists, rheumatologists, critical care team, and nursing protocol committee.

**Fig. 1.**
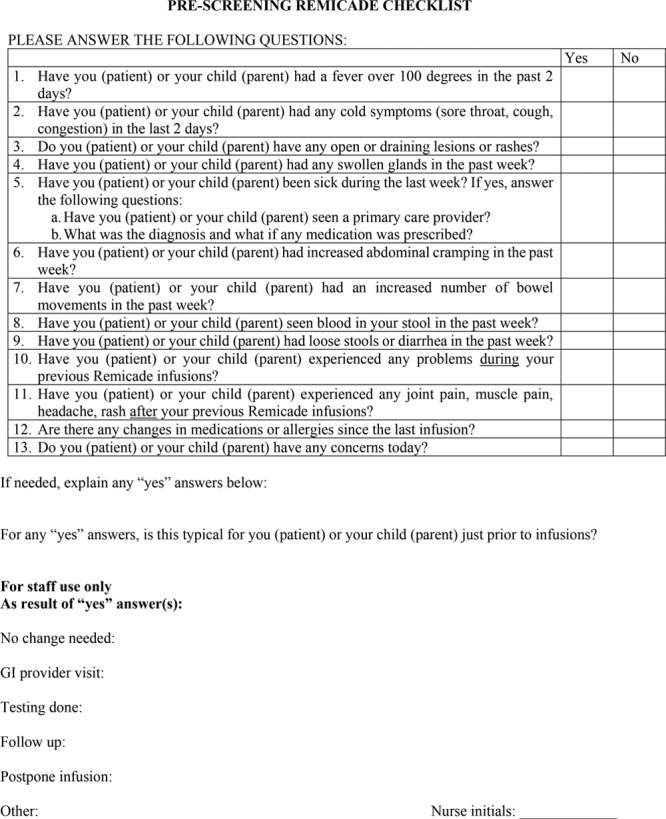
Prescreening safety checklist.

**Fig. 2.**
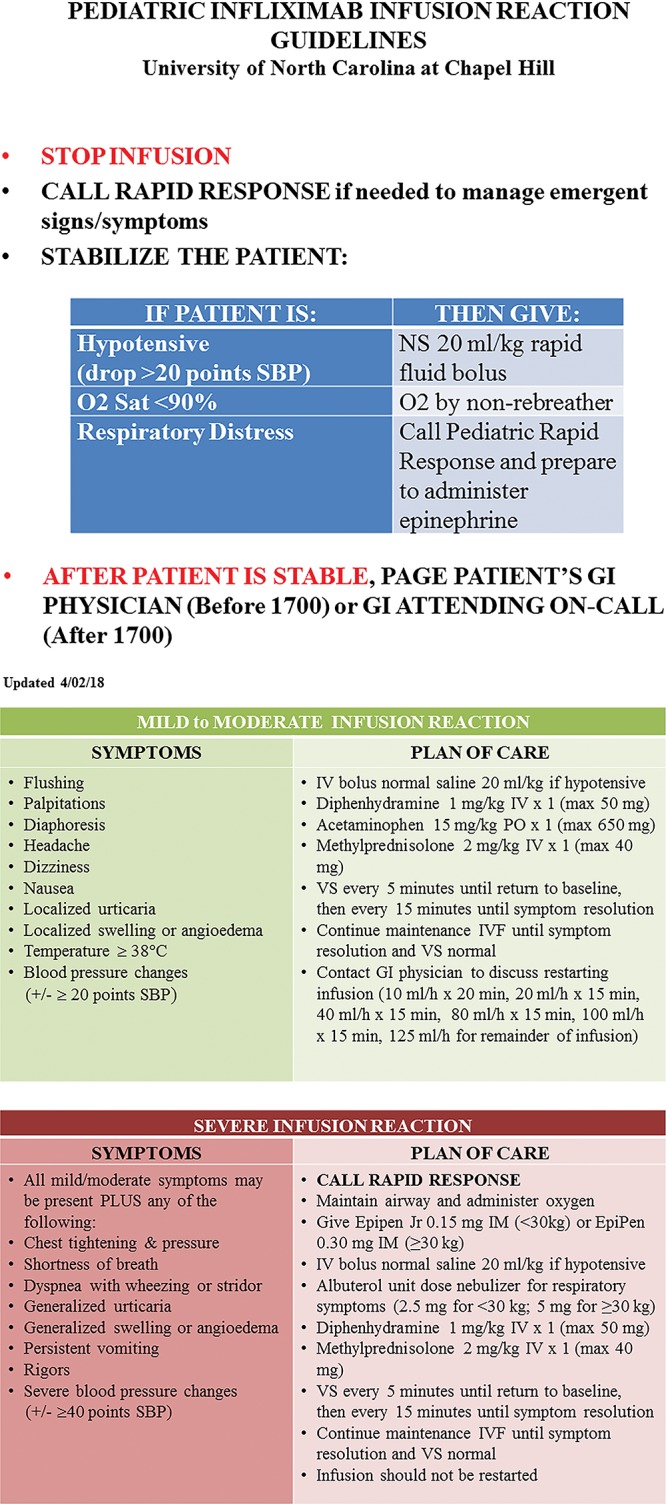
Infliximab infusion reaction guidelines. IV indicates intravenous; max, maximum. SBP, systolic blood pressure; O2 Sat, oxygen saturation; NS, normal saline; PO, by mouth; VS, vital signs; IVF, intraveneous fluids; IM, intramuscular.

The SOP incorporated 6 standards: (1) screening for active infections, worsening disease, and prior infusion reactions with a preinfusion checklist (Fig. 1); (2) laboratory monitoring^2,25^; (3) appropriate administration of the drug (ie, dose, rate of delivery)^17^; (4) parameters for vital sign monitoring^20^; (5) management of infusion reactions^17,19,23^; and (6) premedications and rate adjustments to prevent infusion reactions.^15,17,19,21–23^ The preinfusion checklist, which is the focus of this study, provided data to adjust the plan as needed (eg, postponing the infusion when there was an active infection, increasing the dose for active disease, or administering premedication and slowing down the infusion rate if the patient has had a prior infusion reaction).

Before protocol implementation, the nurse practitioner (NP) led training sessions for all nursing staff in both infusion areas. These sessions included an overview of infliximab, its side effects, and summary of key SOP points. We implemented the SOP in September 2016. We placed a copy on the medication cart in each infusion area. A separate, color-coded guideline outlining the emergency management of infusion reactions was also attached to the medication cart (Fig. 2).

The quality improvement (QI) team members included the NP, physician director, and IR nurse champion. The team made changes to the protocol based on feedback and experience, conducted monthly infusion protocol meetings to discuss and suggest SOP improvements, and shared updates with the nursing staff. The CSSU nurse manager attended QI meetings and disseminated updates to her staff. One gastroenterology (GI) division nurse assisted with changes to the SOP, while another was responsible for gathering and recording data.

### Evaluation: Adherence Outcome Measures

We determined the success of this intervention through fidelity measures to the SOP. This success was assessed primarily by monitoring the submission and completion of the preinfusion checklist following protocol implementation on September 1, 2016 (monitored for 15 months from September 2016 to November 2017). The baseline period was defined as the 6-month post-checklist implementation. A completed checklist required the inclusion of all elements.

The parent completed the checklist on paper for patients under 13 years old; both the parent and patient completed the checklist for patients 13–17 years old. We took this approach so that adolescent patients could begin learning how to answer these questions. For patients 18 years old or older, only the patient completed the checklist. At the end of each week, a designee from each unit brought the completed checklists to the GI division nurse (E.J.L.) who entered them into a deidentified Excel spreadsheet. We completed run chart analyses monthly. The NP reviewed the run charts at monthly infusion protocol meetings.

Although not the focus of our continuous quality improvement activities, we also evaluated the completion of protocol-specified laboratory testing before discontinuation of the intravenous line as an additional measure of protocol fidelity. Unlike the real-time rapid cycle assessment of the checklist completion, the impact of the SOP on laboratory test completion was evaluated using a simple pre–post comparison. For the pre-SOP implementation assessment, we reviewed a priori the records of 50 patients who received infliximab infusions from January 1 to February 12, 2016. This review included all patients who received infliximab over this period. For the post-SOP implementation assessment, we reviewed all patients’ charts for those receiving infusions from August 1 to October 10, 2017 (N = 62). The period for postprotocol data collection was longer than the preprotocol implementation to obtain an adequate sample of patients who had a fourth dose infliximab level drawn. Chart reviews assessed the completion of the 11 laboratory tests specified in the protocol. We compared averages pre- and postimplementation.

### Qualitative Evaluation

**Fig. 3.**
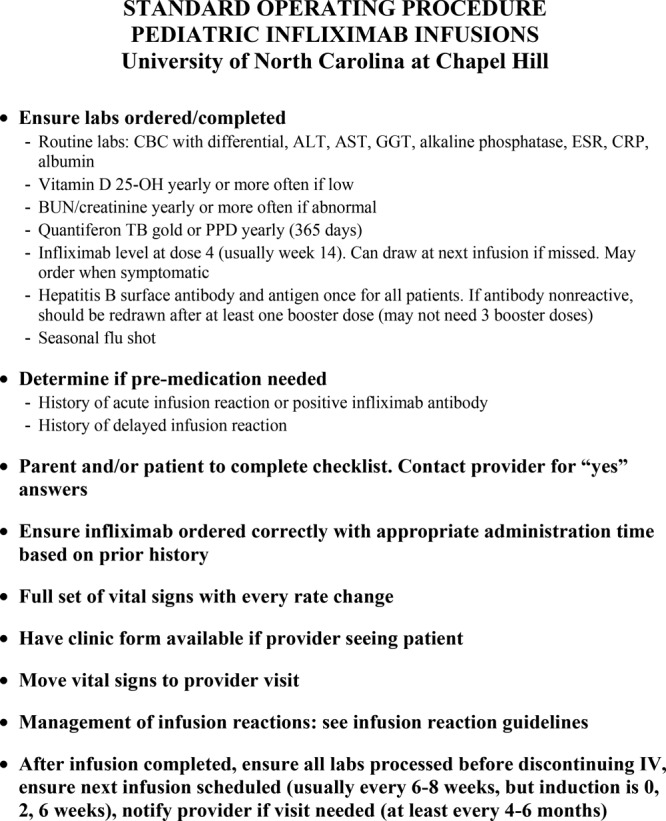
Bulleted version of protocol. ALT indicates alanine aminotransferase; AST, aspartate aminotransferase; BUN, blood urea nitrogen; CBC, complete blood count; CRP, C-reactive protein; ESR, erythrocyte sedimentation rate; GGT, gamma-glutamyl transferase; IV, intravenous; PPD, purified protein derivative; Quantiferon TB Gold, Quantiferon tuberculosis Gold.

During several monthly QI meetings, infusion nurses voiced concerns that the SOP was too complex and lengthy. To address this, the NP simplified the SOP to a 1-page, bulleted guideline (Fig. 3). After incorporating the feedback, the NP conducted 2 nurse training sessions to introduce the condensed protocol, re-explain the purpose of the checklist, and clarify the need for each laboratory test. The revised version of the SOP was placed on the medication cart in the CSSU and IR. The unabridged, original version was also made available for reference as needed.

We requested feedback on the safety checklist in a meeting with 3 nurses and 3 prescribing physicians. The checklist was determined to be too detailed. Therefore, we condensed it by deleting some of the redundant questions. We added a workflow so that the nurses in the infusion units would call the prescriber, document the pertinent positives from the preinfusion checklist, and the response of the provider. Figure 1 represents the final version after iterative improvements.

### Statistical Analysis

We used standard quality improvement methods to evaluate and improve the utilization of the preinfusion checklist. These methods consisted of monthly run charts, stratified by patient care unit. Standard run chart rules were used to interpret the monthly reports.^26^

We evaluated laboratory test completion before and after implementation of the SOP. Independent sample Student’s *t* test (2-tailed) was utilized to compare pre–post adherence for the 8 required laboratory screenings and total laboratory screening. Fisher’s exact test was used to compare the proportion of infusions with testing for tuberculosis, vitamin D, and infliximab level performed at the appropriate time points. All statistical analyses were performed using IBM SPSS Version 24.0.

### Qualitative Measures

The NP conducted focus groups to obtain feedback on the protocol with (1) 6 nurses (2 from CSSU and 4 from IR) and (2) all 4 infliximab-prescribing physicians. She asked 3 open-ended questions during the nurse session:

1. What did you like about the protocol?
2. What was problematic with the protocol or needs improvement?
3. What are your recommendations to sustain the implementation of the SOP?

The same questions were asked during the physician focus group, along with an additional question: What barriers did you find toward the implementation of the protocol? The NP moderated and audio-recorded both sessions. We assessed group engagement by paying attention to body language, eye contact, and facial expressions, as well as each attendee’s level of participation. The NP transcribed the sessions. The responses were coded independently by 2 reviewers (M.M.K., B.S.T.) to reduce subjectivity and bias.

### Ethical Considerations

University of North Carolina (UNC)-Chapel Hill Institutional Review Board reviewed this project and classified it as nonhuman subjects’ research as it involved the establishment of a system-level standard of care.

## RESULTS

### Safety Checklist Submission and Completion

**Fig. 4.**
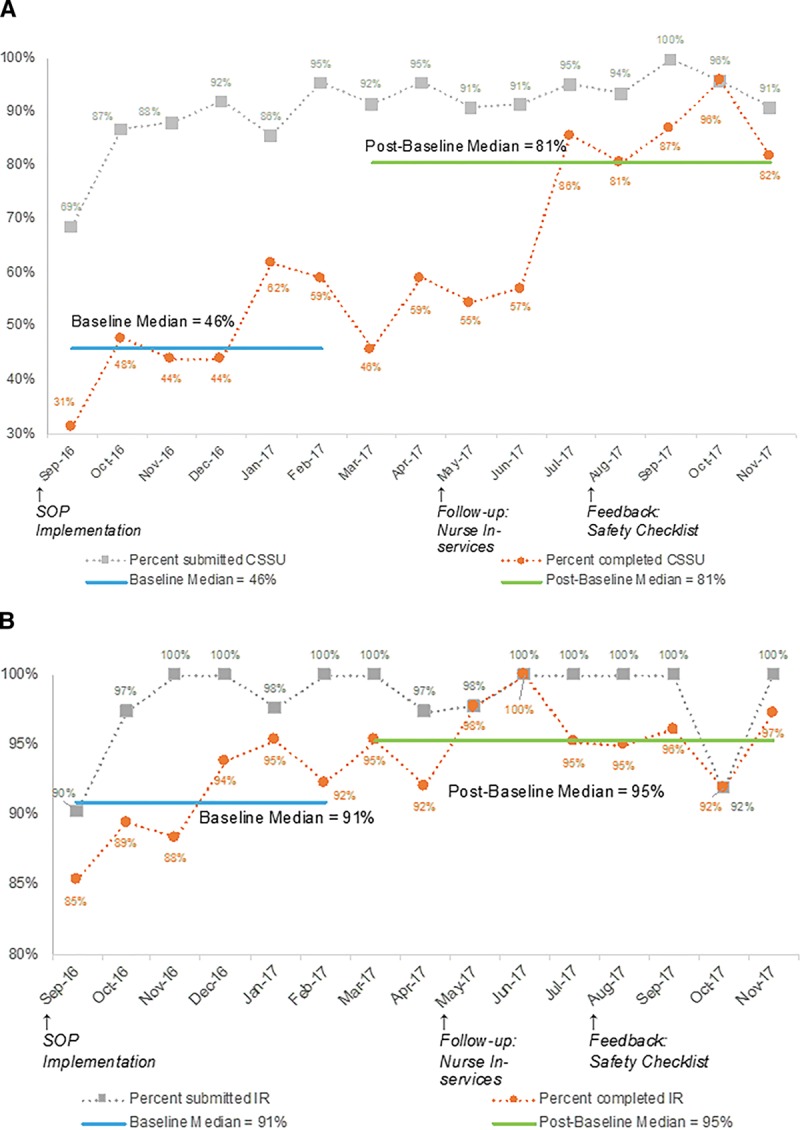
Run charts for fidelity to safety checklist submission and completion. Fidelity to safety checklist submission and completion are shown for (A) CSSU and (B) IR. Note differences in minimum *y* axis value ranges.

The results of the safety checklist submission and completion are displayed in Figure 4. We used standard run chart rules and identified a shift in median completion rates for both units, but no trends or astronomical points.^26^ The baseline period was defined as the 6-month post-checklist implementation. In the CSSU, the median completion rate for the baseline period was 46%, and during the subsequent 9 months, the median completion rate was 81%. In the IR, the median completion rate for the baseline period of 6 months was 91%, and during the succeeding 9 months, the median completion rate was 95%.

### Fidelity/Adherence to Laboratory Screening

Table 1 displays the results comparing pre- and postimplementation fidelity to both standard and periodic laboratory screening. There was a significant increase in the collection of standard laboratory screening (*P* < 0.001), yearly serum vitamin D 25-OH (*P* = 0.032), and fourth dose infliximab levels after implementation of the protocol (*P* < 0.001). Quantiferon (Qiagen, Venlo, The Netherlands) Gold TB or purified protein derivative tuberculosis screening was performed yearly in 100% of patients preintervention and did not decline postintervention; the averages were significantly increased for all but tuberculosis screening (*P* < 0.001).

**Table 1.**
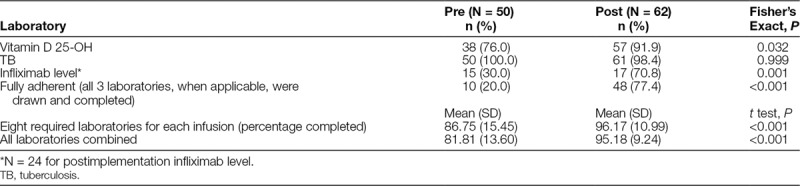
Adherence for Laboratory Screening

### General Focus Group Results

All participants attended the focus groups voluntarily; their facial expressions, the tone of voice, and body language showed engagement. There were no conversation gaps or dominant personalities; each participant had ≥2 responses. All participants were acquainted and communicated well with the moderator and each other. The nurse group stayed on task, without deviation off topic. Initially, the physicians focused specifically on just the checklist; 1 physician helped the NP refocus the group.

### Nurse Focus Group

Twenty-one responses were obtained from the 3 questions posed. The nurses from both infusion areas had consistent responses. Three overall positive themes emerged after 2 independent reviews: communication, quality of care, and efficiency. The nurses agreed that following protocol implementation, there was improved communication between physicians and nurses, patients and nurses, and patients and providers. “Providers seem to be trying to involve nurses more in patient care and not just have us follow orders.” Communication was diminished in 1 case. “Sometimes the provider makes changes to the infliximab dose or interval, and it may be unclear in the EMR (electronic medical record).” Communication was considered essential to sustainability.

The nurses felt that quality of care was improved. “It helped me understand when the patient could have the infusion safely and importance of the checklist.”

Finally, efficiency was felt to be improved by the SOP. “The SOP provided clear guidelines regarding infliximab dilution volumes. This generally results in a smaller IV bag and quicker infusion time.” Efficiency limitations were noted. “It is frustrating to page providers regarding the checklist responses, and they don’t call back quickly.”

### Physician Focus Group

The 4 questions posed to the physicians resulted in 27 responses. Four themes emerged after 2 independent reviews: quality of care, efficiency, communication, and safety.

The physicians agreed that quality of care was improved. The checklist was deemed helpful in decision-making on whether patients should receive their infusion that day. “The infusion nurses are our eyes and ears; a patient may have an infection, or they may not feel well concerning their IBD, requiring a management plan change.”

Overall, efficiency was felt to have improved since SOP implementation. “Monitoring standard labs is helpful.” However, 1 comment suggested efficiency could be improved more. “We should build a lab set into EMR that populates automatically.” One physician voiced that sustainability should not be problematic. “Since biologic infusions are a significant revenue generator, any additional time it takes to use the protocol should be balanced by increased revenue.” There was 1 barrier to sustainability mentioned. “We need to simplify parts that are too complicated for physicians and nurses.”

Communication was felt to be improved overall. “Physician and nurse documentation has improved.” Regarding communication problems causing implementation barriers, “I’m not always available to answer pages. A triage system should be implemented if the nurse doesn’t hear from the provider within five minutes.”

Last, safety was an important theme; “Getting data from the protocol will help improve safety as we start home-based or rapid infusions.” “Management of infusion reactions with an algorithm prevents calling a rapid response or giving epinephrine unnecessarily.”

## DISCUSSION

In this QI initiative, we implemented a facility-developed SOP for infliximab infusions. The project aim was to reduce variations among different nurses, physicians, and infusion units. The SOP and iterative improvements resulted in improved adherence with a preinfusion safety checklist. Monitoring of standard laboratory screening, vitamin D 25-OH, and infliximab levels increased postprotocol.

In regards to the checklist, the IR was more adherent with checklist submission and completion at baseline, but the CSSU showed remarkable progress, and at the end of data collection, their percentage completion was not significantly different from IR. The CSSU showed improvement after the follow-up nurse in-service, but it is not clear if this was the result of the in-service or part of an increased adherence trend unrelated to the in-service. The initial difference in adherence between the units was likely related to the CSSU nurses caring for many types of patients, not just infliximab infusions. Querying CSSU nurses helped uncover why they initially had lower submission and completion rates. Initially, the CSSU nurses assumed the checklist had low importance due to not understanding the rationale. Furthermore, for 13- to 17-year-old patients, nurses did not offer the checklist to both the parent and the patient as they did not understand the purpose of 2 checklists. Education from the nurse manager and NP helped them use the checklist appropriately.

Focus group discussion with nurses revealed major themes of communication, quality of care, and efficiency; focus group discussion with physicians uncovered these same themes, plus safety. Feedback from both groups was mostly positive, and they seemed to feel that quality of care and communication were most improved. Both nurses and physicians were enthusiastic in their willingness to give feedback, likely because they wanted to improve patient care. This feedback helped the QI team understand what was working well and what needed improvement. Nurses seemed to recognize their important role in each patient’s infusion better. Physicians seemed to feel more secure that nurses felt more competent; this improvement was likely due to increased communication between the physicians and nurses.

This project highlights the importance of interdisciplinary collaboration, particularly between nurses and physicians. As the number of patients on biologic therapies continues to grow, consistency in practice is essential. Gaps in education and lack of communication led to many of the inconsistencies at our center. One strength of this project included close monitoring of the SOP which ensured ongoing evaluation as well as continuing opportunity to improve care. For example, we conducted monthly infusion protocol QI meetings after SOP implementation. Initially, this included the NP, physician leader, GI division nurse, and an infusion nurse. However, the group evolved to include a broader group of stakeholders: a nurse manager, hospital QI representative, data manager, rheumatologist, and nephrologist that also prescribe infusions. These meetings focused on what was working well and areas needing improvement. The group is currently collecting additional data that should help improve outcomes.

A limitation of this study is the lack of safety checklist completion data *before* SOP implementation. For this reason, we chose the first 6 months of checklist data after SOP implementation as our baseline. Therefore, we cannot fully determine whether improvement occurred as a result of the SOP and our continuous QI efforts versus a cointervention or secular trend. Additionally, although we utilized continuous quality improvement to optimize the implementation of the checklist portion of the SOP, our continuous QI efforts did not extend to other aspects of the SOP, including laboratory test completion. Rather, we analyzed laboratory testing as a secondary measure of protocol fidelity using a retrospective pre–post analysis. Therefore, formal conclusions about improvement in laboratory testing as a result of SOP implementation cannot be made.

In the future, this SOP will be updated based on new knowledge from the literature and experience with its use. The SOP will also be the basis for creating a protocol for rapid 1-hour infliximab infusions and nonhospital-based infusions, which also require safe, consistent care. It should decrease time away from school and work and enable the units to give infusions to more patients each day. The SOP development process also helped nephrologists and rheumatologists at our center design SOPs for biologics and chemotherapeutic agents. Our pediatric GI division plans to utilize this process to develop protocols for other diseases, such as patients undergoing a liver transplant.

Currently, we only evaluated prescreening safety checklist adherence, laboratory monitoring, and nurse and physician satisfaction. Although quality, safety, communication, and efficiency of patient care were improved, it is unclear whether the protocol has improved patient outcomes. In the future, the infrastructure will be used to measure the impact of the SOP on clinical outcomes, which is essential for determining best practices.

## ACKNOWLEDGMENTS

The authors acknowledge the following members for assistance with the study: Elizabeth Kelly, MBA, for ongoing support, review, and feedback; and Francisco Sylvester, MD, for editing support.

## DISCLOSURE

The authors have no financial interest to declare in relation to the content of this article.
